# Exploring the Bacterial Microbiota of Colombian Fermented Maize Dough “Masa Agria” (Maiz Añejo)

**DOI:** 10.3389/fmicb.2016.01168

**Published:** 2016-07-29

**Authors:** Clemencia Chaves-Lopez, Annalisa Serio, Johannes Delgado-Ospina, Chiara Rossi, Carlos D. Grande-Tovar, Antonello Paparella

**Affiliations:** ^1^Faculty of Bioscience and Technology for Food, Agriculture and Environment, University of TeramoMosciano Sant’Angelo, Italy; ^2^Grupo de Biotecnología, University of San Buenaventura Sede CaliCali, Colombia

**Keywords:** fermented maize, pyrosequencing, *Lactobacillus*, *Acetobacter*, dough

## Abstract

*Masa Agria* is a naturally fermented maize dough produced in Colombia, very common in the traditional gastronomy. In this study we used culture-dependent and RNA-based pyrosequencing to investigate the bacterial community structure of *Masa Agria* samples produced in the south west of Colombia. The mean value of cell density was 7.6 log CFU/g of presumptive lactic acid bacteria, 5.4 log cfu/g for presumptive acetic bacteria and 5.6 og CFU/g for yeasts. The abundance of these microorganisms is also responsible for the low pH (3.1–3.7) registered. Although the 16S rRNA pyrosequencing revealed that the analyzed samples were different in bacteria richness and diversity, the genera *Lactobacillus*, *Weissella*, and *Acetobacter* were predominant. In particular, the most common species were *Lactobacillus plantarum* and *Acetobacter fabarum*, followed by *L. fermentum, L. vaccinostercus*, and *Pediococcus argentinicus.* Several microorganisms of environmental origin, such as *Dechloromonas* and most of all *Sphingobium* spp., revealed in each sample, were detected, and also bacteria related to maize, such as *Phytoplasma.* In conclusion, our results elucidated for the first time the structures of the bacterial communities of *Masa Agria* samples obtained from different producers, identifying the specific dominant species and revealing a complete picture of the bacterial consortium in this specific niche. The selective pressure of tropical environments may favor microbial biodiversity characterized by a useful technological potential.

## Introduction

Maize or corn (*Zea mays*), the “gift of the goddess” of the ancient Amerindians, is a cereal crop that is produced in a wide range of agro-ecological environments worldwide. Archeological evidence from the central Andes indicates that the role of maize changed between A.D. 500 and 1500, shifting from a culinary item, simply prepared by boiling, to a more complex symbolic food with elaborated political meanings, transformed by grinding and chewing into beer (Hastorf and Johannessen, 1993). Today maize plays an extremely important role in the Andean culture and is the main cereal grain in Colombia, as measured by production of 260.700 Ha^1^ (2015).

Fermented foods, besides being more than a pleasure and a satisfaction of nutritional needs, are a rich source of insight into many aspects of cultural life (Hastorf and Johannessen, 1993). Since pre-Hispanic times, fermented products from maize have been consumed widely in Colombia (Chaves-López et al., 2014a), where their manufacturing remains a traditional art in houses, villages, and small-scale industries. In Colombian fermented foods and beverages, microbial interactions have been demonstrated to be of paramount importance in the development of the particular traits of the final product (Chaves-López et al., 2014b).

*Masa Agria*, also known as *Maiz añejo*, is a traditional fermented maize dough, produced in Colombia, which is still prepared by natural fermentation. In the traditional method of preparation, described by Chaves-López et al. (2014a), yellow maize kernels are peeled, covered with water, and stored in a hot place (35 to 40°C) to favor spontaneous fermentation. The period of fermentation varies from 3 to 5 days and determines the degree of sourness with a final pH from 4.4 to 3.8. After fermentation, the grains are washed with water, drained and milled, and then a dough is formed and allowed to stand for 3 or 5 days. During this period, dehydration occurs, and *a*_w_ is reduced at different levels depending on the producers. As reported by the same authors, fermentation is triggered mainly by lactic acid bacteria (LAB) and yeasts that are able to reach values of about 8.8 and 6.8 log CFU/g, respectively. *Masa Agria* is a very common product in Colombian gastronomy, as it is commonly used to prepare soups, tamales (dough filled with meat and vegetables and cooked in a banana leaf), empanadas (cooked dough filled with meat and potatoes and then fried), carantanta (the leftover material in the bottom of the pot that is peeled off after cooked Masa Agria for empanadas) and envueltos (dough steamed in a corn husk).

In the last years, metagenomic analyses have been broadly used to investigate microbial communities of fermented foods (Sakamoto et al., 2011; Chao et al., 2013; Elizaquível et al., 2015; Minervini et al., 2015). In general, these studies revealed a widely unknown microbial biodiversity, underreported by conventional cultivation-based methods. Although several studies have been conducted to explore the bacterial comunities in fermented maize doughs from different countries, to the best of our knowledge no information is available on the microbial communities of the Colombian *Masa Agria*. Therefore, the aim of this study is to explore the microbial diversity of Colombian *Masa Agria* by pyrosequencing of 16S rRNA of the bacterial microbiota, to improve the knowledge on microbial communities that can be essential for product characterisation and process optimization (Oguntoyinbo et al., 2011).

## Materials and Methods

### *Masa Agria* Samples

Six distinct samples of *Masa Agria* from different producers of Valle del Cauca (Colombia) were acquired. Producers were located in the north (producer 1), north-east (2 and 6), and south (3, 4 and 5) of the region. Samples were immediately refrigerated and transferred to the laboratory for the following analyses.

### Microbiological Analyses

Ten grams of each sample were mixed with 90 mL of 0.85% (w/v) sterile physiological saline, and homogenized in a Stomacher Lab-blender 400 Circulator (Seward, Worthing, UK) for 2 min. Serial dilutions were prepared in the same diluent. Total viable count was determined on Plate Count Agar, after incubation at 30°C for 48 h. Sabouraud Dextrose Agar added with Chloramphenicol (Sigma–Aldrich), incubated at 25°C for 72 h, was used for the enumeration of yeasts, while fungi were detected on Czapek Dox Agar after 5 days of incubation at 25°C.

Presumptive LAB were enumerated on Man, Rogosa and Sharpe (MRS) agar and presumptive enterococci on Slanetz and Bartley, both incubated anaerobically by means of anaerobic jars and BBL GasPak anaerobic system envelopes (Becton Dickinson, Cockeysville, MD, USA) at 37°C for 48 h. Micrococci and staphylococci were determined on Mannitol Salt Agar, incubated at 35°C for 48 h. Presumptive *Pseudomonas* spp. were counted on *Pseudomonas* Agar Base added with CFC supplement after 48 h at 25°C. Total coliforms were detected on Violet Red Bile Agar (VRBA) incubated at 37°C for 24 h. Finally acetic bacteria were enumerated on GYC Agar (Conda, Pronadisa, Madrid, Spain) supplemented with 3.0 g/L CaCO_3_ (Sigma–Aldrich, Milan, Italy).

All media were from Oxoid-Thermofisher (Rodano, Italy), except where differently specified. Three repetitions for each sample were performed.

### Physical-Chemical Analyses

The pH value was determined using a pH meter (Mettler Toledo MP 220, Novate Milanese, Italy). Aliquots of 10 g of *Masa Agria* were homogenized thoroughly with 10 mL of distilled water and the homogenate was used for pH determination.

The water activity (*a*_w_) of the samples was determined by means of Aqualab instrument model Series 3 (Decagon Devices, Pullman, WA, US).

Three replicates were analyzed for each sample.

### RNA Extraction from *Masa Agria* Samples and Pyrosequencing Analysis

An aliquot of about 200 mg of each *Masa Agria* sample was diluted in RNAlater (Sigma–Aldrich) and was then used for RNA extraction by Stool total RNA purification kit (Norgen Biotech Corporation, Thorold, ON, Canada). Total RNA was treated with RNase-free DNase I (Roche, Almere, Netherlands; 10 U of DNase for 20 μg of RNA) for 20 min at room temperature. Quality and concentration of RNA extracts were determined by using 1% agarose-0.5X Tris borate EDTA (TBE) gels and by spectrophotometric measurements performed at 260, 280, and 230 nm by using the NanoDrop^®^ ND-1000 Spectrophotometer (Thermo Scientific- Wilmington, DE, USA). Random examers and the Tetro cDNA synthesis kit (Bioline, Taunton, US) were used to transcribe the extracted RNA (about 2.5 μg) to cDNA, according to the manufacturer’s instructions (Gowen and Fong, 2010).

For each dough, three cDNA samples were used for bacterial tag-encoded FLX amplicon pyrosequencing (bTEFAP), which was performed by Research and Testing Laboratories (RTL, Lubbock, TX, USA), using a 454 FLX Sequencer (454 Life Sciences, Branford, CT, USA). The bTEFAP procedures were performed on the basis of RTL protocols http://www.researchandtesting.com (Research and Testing Laboratories, Lubbock, TX, USA). cDNA was analyzed by bTEFAP, using primers forward 28F:GAGTTTGATCNTGGCTCAG and reverse 519R: GTNTTACNGCGGCKGCTG based upon the V1–V3 region of the 16S rRNA gene (*Escherichia coli* position 27–519; Suchodolski et al., 2012). Following sequencing, the QIIME pipeline version^2^ 1.4.0, with default settings, was used to screen, trim, and filter raw sequence data. B2C2^3^) was used to exclude chimeras, according to Gontcharova et al. (2010). Sequences lower than 250 bp were removed. FASTA sequences for each sample, without chimeras, were evaluated.

### Bioinformatics and Data Analysis

The sequences were first clustered into OTUs (operational taxonomic units) clusters with 97% identity (3% divergence) using USEARCH sequence analysis tool^4^ To determine the microbial identities, sequences were first queried using a distributed BLASTn algorithm (Dowd et al., 2005) against a database of high-quality 16S bacterial sequences derived from NCBI. Database sequences were characterized as high quality based on the similar criteria originally described by Ribosomal Database Project (RDP, v10.28; Cole et al., 2009).

Operational taxonomic units were identified using the appropriate taxonomic levels using a database of high quality sequences derived from NCBI.

First, overall richness (i.e., number of distinct organisms present within the microbiome; alpha diversity) was expressed as the number of OTUs, and was quantified using the Chao1 richness estimator:

$$S_{chaol}=S_{obs}+\frac{n1\left(n1-1\right)}{2\left(n2+1\right)}$$

where *n*_i_ is the number of OTU with abundance *i*.

Second, overall diversity (which is determined by both richness and evenness, the distribution of abundance among distinct taxa) was expressed as Shannon Diversity. Shannon diversity (*H′*) is calculated using:

$$H^{\prime }=-\overset{R}{\underset{i=1}{\Sigma }}pi\,\ln\left(pi\right)$$

where *R* is richness and *pi* is the relative abundance of the *i*th OTU.

Venn diagrams were realized on the bases of the OTUs obtained for the different species, according to Heberle et al. (2015).

### Statistical Analysis

All values are shown as means with the standard deviation. The data on microbial population were analyzed by ANOVA. Differences among means were studied using the Tukey’s test at a *p*-value of <0.05, using statistical software STATISTICA 7.0 (Statsoft, Tulsa, OK, USA) for Windows.

As regards pyrosequencing results, the relative abundance of each OTU was determined for each sample and the differences between the samples were calculated using Student’s *t*-test.

Principal Component Analysis was performed to analyze dissimilarities among the samples regarding their bacterial species, using statistical software STATISTICA 7.0 (Statsoft, Tulsa, OK, USA) for Windows.

## Results

### Physical-Chemical Parameters

**Table 1** shows the values of pH and water activity measured in the *Masa Agria* samples. The analyses revealed some differences among samples from different producers. In particular, samples from producer 3 showed the lowest *a*_w_ values, with significant (*p* < 0.05) differences with respect to the other samples (except for sample 6), probably suggesting a more advanced age, thus implying a higher dehydration. In general, pH values of the analyzed *Masa Agria* samples were lower of equal than 3.76, as a consequence of the metabolic activity of LAB and acetic bacteria. Samples obtained by producer 5 were characterized by the lowest pH values, around 3.12.

**Table 1:** Physical-chemical parameters of Masa Agria samples obtained from six different producers.

Producer	pH	*a*_w_
1	3.76 ± 0.01	0.994 ± 0.001
2	3.56 ± 0.02	0.992 ± 0.002
3	3.56 ± 0.01	0.989 ± 0.002
4	3.63 ± 0.02	0.997 ± 0.001
5	3.12 ± 0.02	0.997 ± 0.001
6	3.58 ± 0.03	0.991 ± 0.002


*Data are expressed as mean of three repetitions ± standard deviation.*

### Microbiological Profile of *Masa Agria* Samples

Microbial counts (**Table 2**) revealed a mesophilic aerobic population comprised between 7.4 and 7.9 log CFU/g, with samples 1 and 4 showing the highest counts.

**Table 2:** Microbial counts detected in Masa Agria samples obtained from 6 different producers.

Microbial group	Producer 1	Producer 2	Producer 3	Producer 4	Producer 5	Producer 6
Mesophilic aerobic count	7.87 ± 0.00	7.43 ± 0.05	7.54 ± 0.03	7.90 ± 0.02	7.38 ± 0.02	7.63 ± 0.04
Lactic acid bacteria	8.15 ± 0.03	7.93 ± 0.02	7.56 ± 0.04	7.95 ± 0.02	7.91 ± 0.03	7.93 ± 0.01
Enterococci	2.60 ± 0.02	2.50 ± 0.01	2.76 ± 0.02	2.88 ± 0.02	2.04 ± 0.03	2.01 ± 0.02
Staphylococci	2.03 ± 0.01	<2.00	3.40 ± 0.03	<2.00	3.82 ± 0.03	<2.00
Acetic bacteria	2.48 ± 0.03	3.50 ± 0.04	7.40 ± 0.04	7.80 ± 0.03	7.80 ± 0.05	3.72 ± 0.02
*Pseudomonas*	<2.00	<2.00	2.46 ± 0.01	3.42 ± 0.02	3.75 ± 0.03	2.23 ± 0.03
Coliforms	<1.00	2.48 ± 0.01	<1.00	<1.00	<1.00	2.30 ± 0.04
Yeasts	5.60 ± 0.04	6.60 ± 0.05	5.16 ± 0.03	5.47 ± 0.02	5.13 ± 0.03	5.78 ± 0.03
Molds	2.36 ± 0.02	2.50 ± 0.01	2.76 ± 0.03	2.88 ± 0.04	2.04 ± 0.02	2.43 ± 0.01


*Data are expressed as Log CFU/g (mean of three repetitions ± Standard Deviation).*

Plate counts confirmed that the viable microbiota was dominated by the association of presumptive LAB and yeasts, as already observed by Chaves-López et al. (2014b), together with acetic bacteria, particularly in samples from producers 3, 4, and 5. Counts comprised between 2.0 and 2.9 log CFU/g were observed for presumptive enterococci. The presence of micro-staphylococci was instead variable: while counts of 3.4 and 3.8 were observed in samples 3 and 5, respectively, they were undetectable (<2.0 log CFU/g) in samples 2, 4, and 6.

Significantly different numbers (*p* < 0.05) were counted for presumptive *Pseudomonas* spp. in the different samples, with counts below 2.0 log CFU/g only in samples 1 and 2. Coliforms were detected only in samples 2 and 6.

### Sequencing Statistics and Diversity Estimate

Pyrosequencing of the bacterial 16S rRNA genes generated more of 33.800 reads for each samples. The optimized data at 97% sequence similarity cut-off are shown in **Table 3.** The bacterial community was analyzed by richness estimator (Chao 1), and diversity index (Shannon). In general samples from producers 2 and 6 harbored higher bacterial diversity (Shannon index) than the other samples and similar patterns of richness (Chao 1), as shown in **Table 3.**

**Table 3:** Comparison of estimates OTUs, richness and diversity indices of the 16S rRNA gene libraries as obtained from the pyrosequencing analysis.

	Number of Reads	Number of OTUs	Chao 1	Shannon
Producer 1	34076	15	15	1.48
Producer 2	34001	28	25	2.27
Producer 3	33947	18	9	1.45
Producer 4	34006	18	8	1.23
Producer 5	34105	15	9	1.38
Producer 6	33862	33	25	2.21


### Microbial Community Structure

Taxonomy-based analysis showed a total of seven bacteria Phyla (*Bacterioidetes, Actinobacteria, Cyanobacteria, Firmicutes, Proteobacteria, Planctomycetes*, and *Tenericutes).*

**FIGURE 1:**
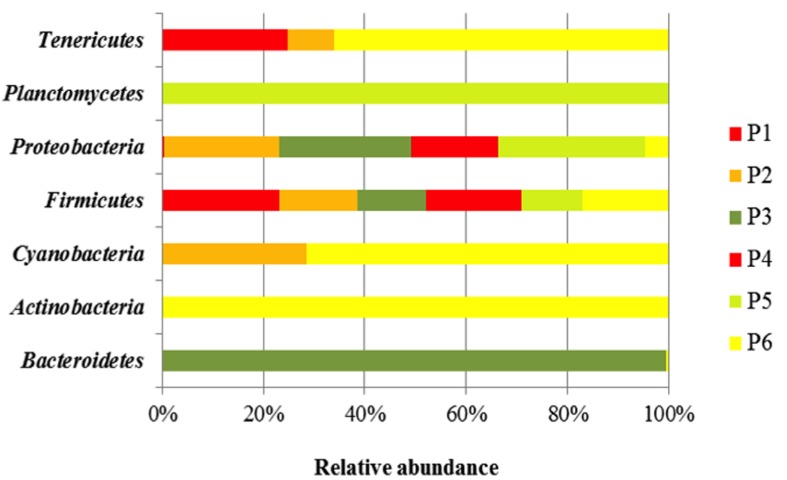
**Bacterial community diversity in six *Masa agria* samples from different producers (P, producers).** The relative abundance of bacterial 16S rRNA genes was estimated through classification at the Phyla level.

*Firmicutes* was the major Phylum in the different fermented doughs, containing for more than 47% of all the bacterial community (**Figure 1**). The relative abundance of *Proteobacteria*, detected in each sample, was lower than *Firmicutes*, in particular in the samples from producers 1, 2, and 3, while Phylum *Tenericutes* was found only in samples of producers 1, 2, and 6. *Bacteriodetes* and *Planctomycetes* were present only in samples from producers 3 and 6, respectively.

In **Table 4** the relative abundance of microbial components at Family level in *Masa Agria* doughs is reported. *Firmicutes* were divided in three families (*Lactobacillaceae, Leuconostocaceae*, and *Streptococcaceae*), with *Lactobacillaceae* as the most abundant, accounting for a minimum of 28.5% (producer 3) to 89% (producer 1), followed by the sequences attributed to *Leuconostocaceae*, up to 18.2% (producer 2), and 20.1% (producer 6). *Streptococcaceae* were present only in samples from producers 2 (3.7%), 4 (0.04%), and 6 (6.2%).

**Table 4:** Bacterial community diversity in six samples of *Masa Agria* from different producers (*P*, producer).

Family	P1	P2	P3	P4	P5	P6
*Flavobacteriaceae*	-	-	0.20	-	-	0.03
*Streptomycetaceae*	-	-	-	-	-	2.15
*Bacteroidaceae*	-	-	0.09	-	-	-
*Lactobacillaceae*	89.38	29.64	28.52	29.69	33.04	39.14
*Leuconostocaceae*	0.10	18.17	0.45	0.27	0.03	20.11
*Streptococcaceae*		3.70	-	0.04	-	6.27
*Acetobacteraceae*	0.36	4.50	69.94	69.49	65.54	5.82
*Sphingomonadaceae*	0.20	0.50	0.22	-	0.41	1.0
*Rhodocyclaceae*	-	-	0.02	-	0.03	-
*Comamonadaceae*	-	0.08	-	-	-	0.17
*Moraxellaceae*	-	0.08	-	-	-	0.07
*Enterobacteriaceae*	-	0.26	-	-	-	0.41
*Pseudomonadaceae*	-	-	0.07	-	0.47	0.06
*Thiotricaceae*	-	-	0.33	-	0.31	-
*Xanthomonadaceae*	-	0.043	-	-	-	0.07
*Planctomycetaceae*	-	-	-	-	0.09	-
*Acholeplasmataceae*	9.91	40.35	-	-	-	23.49
Unknown	-	0.04	-	-	-	0.06
Unclassified	-	-	-	-	-	0.1


*The relative abundance of bacterial 16S rRNA genes was estimated through classification at the Family level.*

*Proteobacteria* Phylum was represented by nine families: *Acetobacteriaceae, Sphingomonadaceae, Rhodocyclaceae, Coma monadaceae, Enterobacteriaceae, Moraxellaceae, Xanthomona daceae, Thiotrichaceae*, and *Pseudomonadaceae*. In particular *Acetobacteriaceae* family was present in all the samples and was the most abundant family, accounting for 100% of *Proteobacteria* in sample from producer 4. Except for samples 4 and 1, in which only two families where represented (*Acetobacteraceae* and *Sphingomonadaceae*), OTUs of at least five families were present.

*Planctomycetes* Phylum, that was only a smaller proportion of the samples profile, was represented only by *Planctomycetaceae* family, with a incidence of 0.09% in sample from producer 3. *Tenericutes* Phylum was represented by the *Acholeplasmataceae* family that accounted for 9.91, 40.35, and 23.5% in samples from producers 1, 2, and 6, respectively.

At genus level, the number of identified OTUs of the samples varied depending on the producer: the samples from producers 2 and 6 were characterized by the major presence of OTUs, with 14 and 21 different genera, respectively, while in the other samples 6, 11, 6, and 8 OTUs were revealed (in samples from producers 1, 3, 4, and 5, respectively), as described in **Table 5.** The top 9 most abundant OTU demonstrated that *Lactobacillus*, belonging to *Firmicutes* Phylum (ranging from 17.3 to 89.3%), dominated in all the samples followed by *Acetobacter* (*Proteobacteria* phylum; from 0.36 to 69.9%) and *Weissella* (from 0.03 to19.1%) (**Table 5**).

**Table 5:** Bacterial community diversity in *Masa Agria*.

Genus	P1	P2	P3	P4	P5	P6
*Bacteroides*	-	-	0.08	-	-	-
*Streptomyces*	-	-	-	-	-	0.07
*Chryseobacterium*	-	-	0.20	-	-	0.03
*Planktothricoides*	-	-	-	-	-	0.10
*Lactobacillus*	89.33	26.86	17.35	26.80	33.03	37.30
*Pediococcus*	0.051	2.78	11.17	2.87	-	3.83
*Leuconostoc*	-	0.91	0.22	-	-	0.97
*Weissella*	0.10	17.28	0.22	0.27	0.03	19.13
*Lactococcus*	-	3.78	-	0.03	-	6.20
*Streptococcus*	-	-	-	-	-	0.06
*Gemmata*	-	-	-	-	0.09	-
*Acetobacter*	0.36	4.57	69.93	69.33	65.54	6.92
*Sphingomonas*	-	-	-	-	-	0.03
*Comamonas*	-	0.08	-	-	-	0.03
*Enterobacter*	-	0.17	-	-	-	0.12
*Delftia*	-	-	-	-	-	0.14
*Escherichia*	-	0.04	-	-	-	0.27
*Serratia*	-	0.04	-	-	-	0.03
*Acinetobacter*	-	0.08	-	-	-	0.06
*Frateuria*	-	0.04	-	-	-	-
*Gluconobacter*	-	-	-	0.015	-	-
*Sphingobium*	0.23	0.52	0.223	-	0.41	1.01
*Dechloromonas*	-	-	0.22	-	0.03	-
*Pseudomonas*	-	-	0.06	-	0.47	0.06
*Thiothrix*	-	-	0.33	-	0.03	-
*Stenotrophomonas*	-	-	-	-	-	0.07
*Candidatus Phytoplasma*	9.91	40.35	-	-	-	23.49


*The relative abundance of bacterial 16S rRNA genes was estimated through classification at the genera level.*

*Pediococcus* was present in all samples with the exception of sample from producer 5. Several sub-dominants genera (less than 4%), belonging to Proteobacteria (*Comamonas, Enterobacter, Escherichia, Serratia*, and *Acinetobacter*), were identified only in samples from producers 2 and 6. *Sphingobium* was detected in all samples excepted for sample 5. *Dechloromonas*, and *Thiothrix* were found in the samples from producers 3 and 5, while *Pseudomonas* was also found in samples from producer 6. *Gemmata* was found only in sample 5.

The analysis on the species level showed that a certain percentage of the readers could not be classified to any existant group, which is common in pyrosequencing, because many uncultured bacteria can be detected or because they represent new species. The total number of OTUs at species level was 56, of which 15, 27, 18, 18, 15, and 33 were detected in the samples from producers 1 to 6.

**FIGURE 2:**
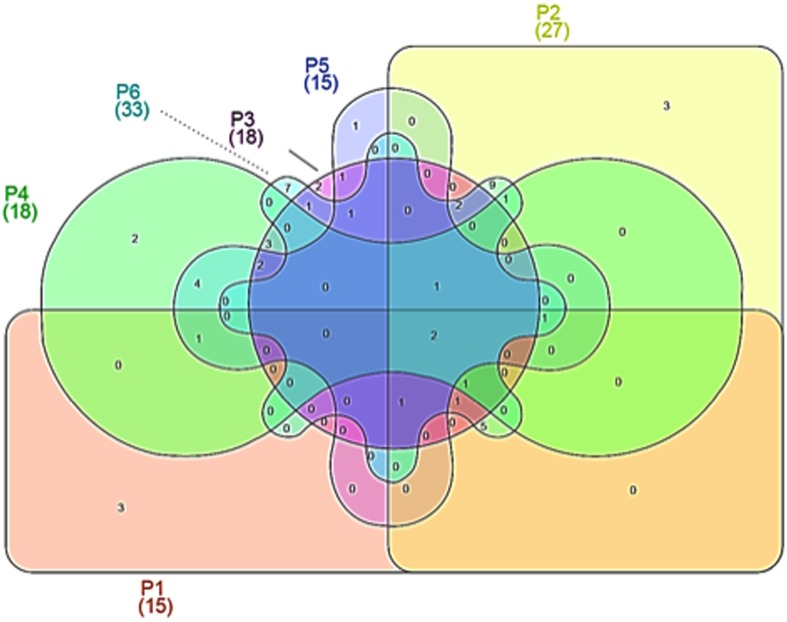
**Venn diagrams showing operational taxonomic units (OTUs) at species level, depicting similarities and differences among in the microbial community in six *Masa agria* samples from different producers (P, producers)**.

Venn diagrams were plotted in order to evidence similarities among different microbial communities in the different samples, in terms of OTUs overlapping (**Figure 2**). As evidenced, only 2 OTUs were shared by all maize dough samples. The number of shared OTUs among the dough producers was low; for example, 11 between doughs from producer 1 and producer 2, while 8 between doughs from producer 2 and producer 3. Doughs from producers 2 and 6 shared the greatest number of OTUs, with 24 common species. Number and abundance of OTUs from rRNA samples are reported in **Supplementary Table S1**, with taxonomic details up to species level when such assignment was possible.

**FIGURE 3:**
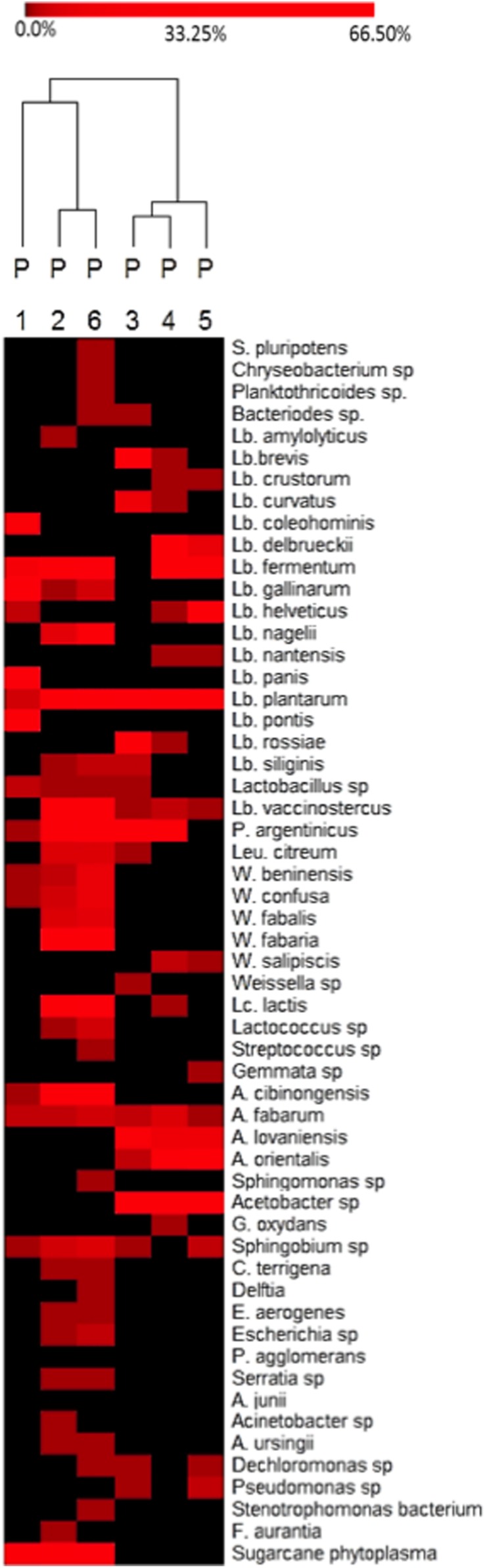
**Heat-map depicting bacterial diversity and relative abundance of species in six *Masa agria* samples from different producers (P, producers), as revealed by 16S rRNA pyrosequencing.** The color key defines the percentages of OTUs in the samples.

To analyze bacterial community at species level among the different producers, a heat-map plot was generated using the relative abundance of the OTUs (**Figure 3**). As evidenced, the dominant OTUs in doughs from producers 1, 2, and 6 were different from those of the producers 3, 4, and 5. In fact in dough from producer 1, *L. gallinarum* (62%) was dominant, *Sugarcane phytoplasma* (40%) in dough from producer 2, and *L. fermentum* in dough 6 (19%), while doughs from producers 3, 4, and 5 were dominated by species of the genus *Acetobacter*, such as *Acetobacter cibinongensis, A. fabarum, A. lovaniensis*, and *A. orientalis*. It has to be underlined that the producers 1, 2, and 6 are located in the north/north-east of the region, while the others were located in the south, thus suggesting differences in the environmental conditions (i.e., temperature, water, and maize provenance).

*Lactobacillus plantarum* and *A. fabarum* were the most common species in all the samples, independently on the producers.

**FIGURE 4:**
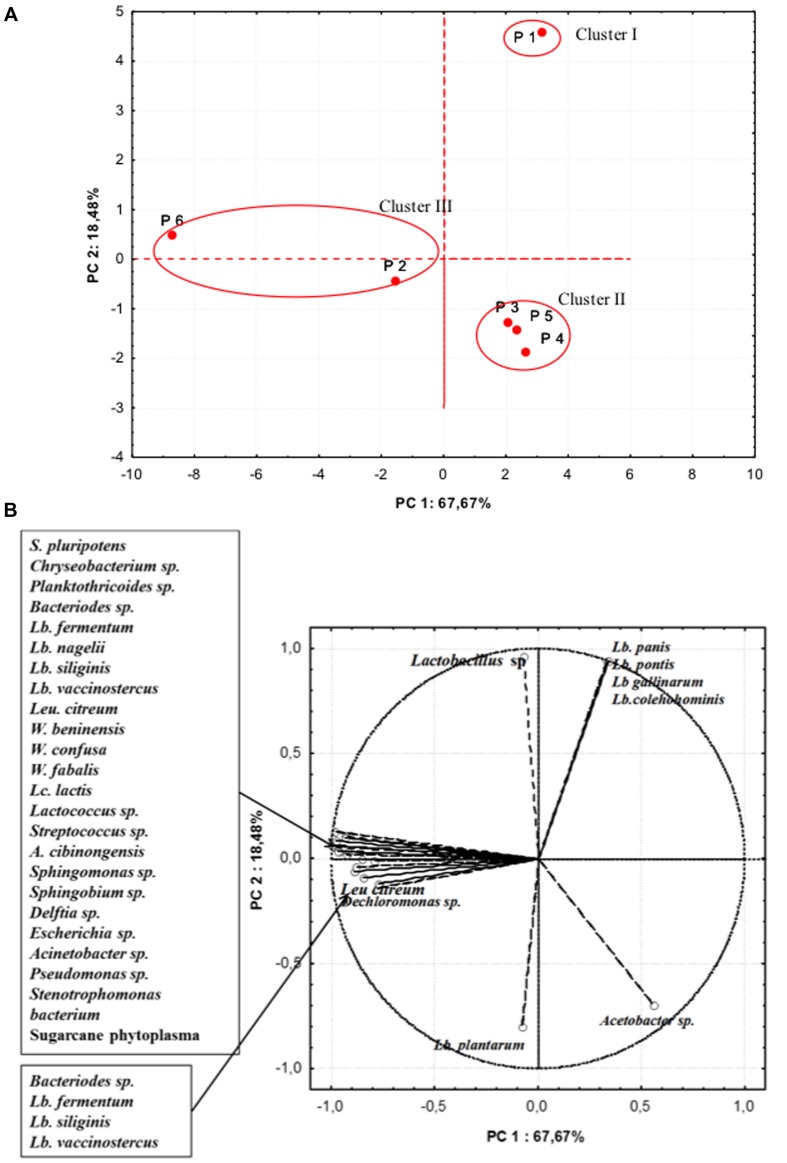
**Principal component analysis (PCA) on the relative abundance of bacterial species.** **(A)** Scores **(B)** Loadings.

In further analyses, aimed at highlighting the microbiota similarity among the different producers, a PCA on the realtive abundance of bacterial species was performed (**Figure 4**). The PC1 accounted for the 67.67% of the variance while PC2 for 18.48%. Samples were separated in three cluster: cluster I containing sample from producer 1, was distinguished by the relative high percentages in OTUs of *L. pontis, L. panis, L. gallinarum, L. coleohominis*, and *Lactobacillus* spp. Cluster II was formed by samples from producers 3, 4, and 5, that were characterized by a relative high abundance of *L. plantarum* and *Acetobacter* spp. The samples from producers 2 and 6 were included in cluster III, featuring a high relative abundance of OTUs of *Lactobacillus* species, *Weissella fabaria* and *S. phytoplasma*, and contained also unique species, probably due to different geographical origin of maize and producers.

## Discussion

In this preliminar study, we used the pyrosequencing of tagged 16S rRNA gene amplicons to explore the bacterial microbiota in Colombian maize fermented dough *Masa Agria*. The data here presented provide a detailed insight of the bacterial profile of this product, as in our knowledge this is the first report of the bacterial community and structure of this Colombian fermented dough.

Microbial culture revealed growth on the plate counts media used. While the presence of presumptive *Lactococcus, Lactobacillus*, acetic bacteria and *Pseudomonas* was confirmed by 16S rRNA pyrosequencing, micro-staphylococci and enterococci were not confirmed. Enterococci may derive from raw materials, such as maize kernels or water, from the environment, and also from the tools used during grain milling and dough manufacturing (Elizaquível et al., 2015). Being able to grow over a wide range of temperature and easily supporting acid pH (Giraffa, 2002; Serio et al., 2010), their presence in the fermented *Masa Agria* would be unsurprising. Nevertheless our *Masa Agria* samples were market samples, analyzed at the end of the fermentations process, therefore enterococci deriving from raw materials and environment could have been overgrown by LAB species, as it happens in sourdough, where they are usually found in the first days (De Vuyst et al., 2014). Some authors reported that Slanetz and Bartley Agar lacks of selectivity when used for food samples, as it could over-estimate the actual number of enterococci, being able to support the growth also of lactococci and some *Lactobacillus* strains (Klein and Reuter, 2012). On the other side, also Mannitol Salt Agar was demonstrated to give false positive results as regards staphylococci, therefore lacking of specificity (Kircher et al., 2002). *Pseudomonas* spp. were present at extremely low sequence reads; indicating that the counts of bacteria that grew on the medium used (PSB + CFC) were unlikely to be all *Pseudomonas.* In fact, although the used medium is selective for this genus, the growth of other Gram-negative bacteria such as *Serratia marcescens, Aeromonas* spp., *Xanthomonas* spp., *Alcaligenes* spp., and *Acinetobacter* spp. is possible (Krueger and Sheikh, 1987).

The presence of lactobacilli, acetic bacteria and yeasts explains the low pH values profiles of the analized doughs from six different producers. The pH values observed in *Masa Agria* samples, are compatible with those reported in literature for other maize doughs (Annan et al., 2003). Chaves-López et al. (2014b) reported the presence of lactic and acetic acids in *Masa Agria* samples obtained from the same geographic area in Colombia. These acids not only are responsible for the sour taste, but their effect in pH reduction play a key role in the activation of endogenous and bacterial phytases, increasing dough nutritional value. In fact, phytic acid complexes amino acids and minerals, therefore acting as anti-nutritional factor. High LAB abundance and low pH are also responsible for coliforms decrease (Elizaquível et al., 2015).

Our findings showed that complex microbiota is associated to natural fermented maize doughs and that community membership and structure considerably differed depending on the producer. In fact, some OTUs were detected only in samples from one producer; in addition, the bacterial composition changed in terms either of species and of their relative abundance. This is not surprising, because the bacterial composition of fermented doughs differed on the bases of ecological parameters such as type of maize used, temperature, time of fermentation, water activity, etc. Another possible explanation for differences among the samples could derive from phytochemical treatments that the maize was subjected to, during the cultivation. In fact, the total microbial population and the relative species proportion on maize grains can be affected by many factors, mainly temperature and rainfall, physical damage due to insects and application of phytopharmaceuticals. Although the producers of the analyzed samples of *Masa Agria* in this study did not know the provenience of the maize, in Colombia it is cultivated from 350 to 2400 meters above sea level. Thus it is evident that bacteria from this natural environment are different and their growth during the fermentation process lead to particular treats of each *Masa Agria*.

Interestingly, 4% of the readings could not be associated with a known species suggesing that *Masa Agria* may be an unexplored reservoir of unknown bacterial species.

In different *Masa Agria* many bacterial species have been found, and some of them have been commonly reported in indigenous Mexican or African fermented maize dough, considerably contributing to the development of the final characteristics of the products (Ben-Omar and Ampe, 2000; Escalante et al., 2001; Abriouel et al., 2006; Assohoun-Djenia et al., 2016). However, the predominant bacterial consortium depends on its source of production with mixtures of maltose and non-maltose fermenting species. As regards the bacterial active population in *Masa Agria*, evidenced by 16S rRNA pyrosequencing, it is immediately clear that LAB, and particularly *Lactobacillus* spp. and *Weissella* spp., together with *Acetobacter* spp., were dominant in all the samples. In particular, while several differences were observed among samples from the different producers, the two species *L. plantarum* and *A. fabarum* were common in all the analyzed doughs. The sequences obtained in the samples from the different producers exhibited high similarities and the species *L. fermentum, L. vaccinostercus, P. argentinicus*, and *Sphingobium* spp. were repeatedly found. This observation suggests that these particular species could play a specific role in *Masa Agria* production and that they could be a typical microbiota of this type of maize fermented product, although the variability should be deeply investigated.

In Mexican fermented maize dough Pozol, Escalante et al. (2001) reported the dominance of *Lc. lactis* followed by *L. alimentarius*, *L. plantarum*, and *Streptococcus suis.* In addition, Ben-Omar and Ampe (2000), studying the bacterial succession during its production, at the end of the fermentation detected *L. plantarum*, *L. fermentum*, *L. casei*, *S. bovis*, *Bifidobacterium minimum*, or *Exiguobacterium aurantiacum*. Halm et al. (1993) concluded that a homogenous group of obligatively heterofermentative lactobacilli related to *L. fermentum* and *L. reuteri* played a dominating role during the production of Ghanaian maize dough *Kenkey*. On the other hand, the analysis of bacterial community composition of maize dough samples from the Congo Republic, by 16S rRNA gene temporal temperature gradient gel electrophoresis (TTGE), revealed that the most intense band corresponded to *L. plantarum*/*paraplantarum;* moreover, although other bacteria such as *L. gasseri*, *Enterococcus* spp., *L. delbrueckii*, *L. reuteri*, *L. casei*, *L. acidophilus*, *L. delbrueckii*, *E. coli*, and *Bacillus* spp. were detected, they were represented by DNA bands of lower intensities (Abriouel et al., 2006). Recently Assohoun-Djenia et al. (2016) reported the predominance of *L. fermentum, L. plantarum, P. pentosaceus, P. acidilactici*, and *W. cibaria* species in *Doklu* from Côte d’Ivoire.

The selective pressure of tropical environments may favor microbial biodiversity and highlights a useful technological potential (Chaves-López et al., 2009), and the geographical isolation among the fermented maize dough products leads to great divergent microbial communities, agreeing with the fact that each maize fermented dough can be considered as unique. Nevertheless it is evident that *L. plantarum* and *L. fermentum* are important species in fermented maize dough. Kunene et al. (2000) suggested that *L. plantarum* and *L. fermentum* associate in spontaneous fermentation of cereals-based foods.

The importance of *L. fermentum* in maize fermentation has been confirmed by previous researches in Ghanan, Benin, Mexican fermented maiz doughs (Agati et al., 1998; Ampe et al., 1999; Hayford et al., 1999; Oguntoyinbo et al., 2011; Obinna-Echem et al., 2014) and the high amylolitic activities found in different strains suggest that *L. fermentum* may be a key organism for fermentation of maize, making the large amounts of starch available to the overall community. In addition, the fermentation products (lactate, formate, and ethanol) may also serve as carbon sources for organisms, such as yeasts (Ben-Omar and Ampe, 2000). Interestingly, Assohoun-Djenia et al. (2016) reported a high prevalence of bacteriocin-producing *L. fermentum* strains, and their detection in different stages of *Doklu* production indicates a high potential of these strains to grow and dominate the microbial population in the fermented maize dough.

The amylolytic activity of some strains of *L. plantarum, L. lactis, Streptococcus* spp. and *Leuconostoc mesenteroides* had been also reported (Sanni et al., 2002; Díaz-Ruiz et al., 2003). In particular the presence of amylolytic *L. plantarum* in cereal fermented products is associated to (i) increasing the availability of energy sources for other associated non-amylolytic LAB (ii) contributing to a rapid pH decrease, and (iii) imparting favorable rheological properties to the dough (Sanni et al., 2002). *L. plantarum* is considered a highly acid-tolerant LAB that dominates in fermentation processes with vegetables and cereals, due to its metabolic flexibility and low pH adaptation (Vrancken et al., 2011). Also several strains of this species have been reported to display a broad spectrum of anti-fungal activity (Schnürer and Magnusson, 2005). Thus, it is possible that these features (amylolytic activity, acid tolerance, and bacteriocin production) contributed to the consolidated presence of both *L. plantarum* and *L. fermentum* in maize fermented dough.

*Acetobacter*, which was the second most represented genus in *Masa Agria*, varied in abundance among the samples, and was particularly abundant in those from producers 3, 4, and 5. Members of this genera are obligately aerobic bacteria that oxidize ethanol to acetic acid, although some species are also able to further oxidize acetic acid completely to CO_2_ and water (Hutkins, 2006). Different species of the genus *Acetobacter* have been associated with whole crop maize silage (Oude Elferinck et al., 2001), where they dominate the first step of fermentation (Sträuber et al., 2012), together with LAB. While the presence of acetic acid bacteria in naturally fermented wheat dough, such as sourdough, is uncommon, on the contrary *Gluconobacter oxydans* and *A. xylinum*, together with *L. saccharolyticum* and *Saccharomyces cerevisiae* have been demonstrated to be important in the fermentation of sorghum grains to produce Hussuwa (El Nour et al., 1999). *Acetobacter* genus has been also associated with maize doughs (Ampe et al., 1999).

Dough fermentation leads to selective environmental conditions, due to sugar consumption and to the progressive pH reduction. Being acid-tolerant, and utilizing also molecules other than sugars for their energetic needs, *Acetobacter* spp. could be selected in the later stage of fermentation. In a polyphasic study on spatial distribution of microorganisms in *Pozol* from Mexico, Ampe et al. (1999) reported the presence of yeasts, fungi, EPS producers (including members of the genus *Leuconostoc*), and enterobacteria, as well as other non LAB, such as members of the genus *Acetobacter*, at the periphery of a pozol ball, in the outer part. Thus it can be hypothesized that samples of producers 3, 4, and 5 were collected overall from the surface of the dough, where oxygen should not have been a limiting factor for the growth of this genus, while the low presence found in samples from producers 1, 2, and 6 could probably be related to the poor presence of *Acetobacter* inside the dough. Further studies on spatial distribution of microorganisms in *Masa Agria* should be performed to confirm this hypothesis.

The presence of *Proteobacteria* observed in *Masa Agria* samples, such as *Comamonas, Sphingomonas, Acinetobacter*, and *Pseudomonas* spp., has been also recognized in the first step of rye and durum wheat sourdough fermentation (Ercolini et al., 2013), as flour and environment contaminants. In sourdough these genera usually become dominated by LAB such as *Lactobacillus* and *Weissella* in the following fermentation steps. However, it has to be underlined that *Masa Agria* production does not imply refreshments as in the case of sourdough, but the fermentation first of maize kernels and then of maize dough, therefore determining different dynamics of microbial succession.

Girma et al. (1989) observed that *Pseudomonas aeruginosa* inoculated in the Ethiopian fermented bread Tef injera, grew well until dough pH was reduced to 5.5, and thereafter the population decreased until only few viable cells were isolated at pH 4.0. Nevertheless, *Pseudomonas* species are characterized by a wide metabolic adaptability to substrates and stressing conditions, thus peculiar species could be selected by the *Masa Agria* environment. On the other hand, *Enterobacteriaceae* such as *Escherichia, Serratia* spp., and *Enterobacter aerogenes* which could derive from the maize kernels, but more probably from the water used for the dough, were only recognized in samples from producers 2 and 6. It is noteworthy the absence of enteropathogenic species.

Several species of environmental origin were recognized in the different samples and particularly notable are *Sphingobium* spp. and *Dechloromonas* spp., playing a role in soil and water bioremediation (Young et al., 2007), and *Gemmata* spp. (sample 5), a freshwater bacteria originally isolated from Queensland. The presence of *Sphingobium* spp. is correlated with the water used to wet the maize during *Masa Agria* production, as it has the capacity to survive in chlorinated waters, allegedly due to the oligotrophic character and the production of biofilms (Vaz-Moreira et al., 2011). This species has been involved in the degradation of chloroacetamide herbicide butachlor (Kim et al., 2013). On the other hand, the relative abundance of *Phytoplasma*, a plant pathogen able to cause maize bushy stunt that is among the most widespread diseases in herbaceous hosts, causing severe yield losses, may be assumed by the fact that the dough samples from producers 1, 2, and 6 were presumptively obtained from maize of low quality.

The complex microbiota of *Masa Agria* included also some less-abundant species such as some *Lactobacillus* (*curvatus, rossiae*, and *silingis*), and *L. citrineum* that migh play an important role in effectiveness and stability of the microbial community, as their microbial metabolism provides molecules able to affect this food ecosystem.

The combined application of culture-dependent and culture-independent analytical strategies allowed us to obtain an insight on the richness of the microbiota of *Masa Agria*, providing information on the diversity and on the relative abundance of microbial species.

## Conclusion

For the first time, this study explored the microbial diversity of *Masa Agria*, by pyrosequencing of 16S rRNA gene amplicons. Our results elucidated the structures of the bacterial communities of six samples obtained from different producers, identified specific dominant species, and suggested the presence of possibly unknown microorganisms.

In particular, this research was focused on bacterial characterisation at the end of fermentation in commercial samples. Further investigations are needed to evaluate the microbial dynamics throughout the manufacturing process and to investigate the role of the different bacterial groups during fermentation.

## Author Contributions

CL: Devised and drafted the manuscript-statistical analyses. AS: drafted the manuscript. JO: molecular analysis. CR: culture dependent analyses. CT: statistical analyses. AP: manuscript revision.

## Conflict of Interest Statement

The authors declare that the research was conducted in the absence of any commercial or financial relationships that could be construed as a potential conflict of interest.

## Footnotes

1 www.dane.gov.co

2 http://qiime.sourceforge.net

3 http://www.researchandtesting.com/B2C2.html

4 http://drive5.com/usearch.
