# Supplementary material for: Exploring the Bacterial Microbiota of Colombian Fermented Maize Dough “Masa Agria” (Maiz Añejo)
Source: Front Microbiol. 2016 Jul 29;7:1168. doi: 10.3389/fmicb.2016.01168 (PMC4965452; doi:10.3389/fmicb.2016.01168)
Supplement: Supplementary file 1 [file Table_1.DOCX]

**Table S1.** Bacterial community diversity in *Masa Agria*. The relative abundance of bacterial 16S rRNA genes was estimated through classification at the species level.

|  | P 1 | P 2 | P 3 | P 4 | P 5 | P 6 |
| --- | --- | --- | --- | --- | --- | --- |
| *S. pluripotens* | 0 | 0 | 0 | 0 | 0 | 0.069711 |
| *Chryseobacterium sp* | 0 | 0 | 0 | 0 | 0 | 0.034855 |
| *Planktothricoides sp.* | 0 | 0 | 0 | 0 | 0 | 0.104566 |
| *Bacteriodes sp.* | 0 | 0 | 0.089206 | 0 | 0 | 0.243987 |
| *Lb. amylolyticus* | 0 | 0.08707 | 0 | 0 | 0 | 0 |
| *Lb.brevis* | 0 | 0 | 4.014273 | 0.038911 | 0 | 0 |
| *Lb. crustorum* | 0 | 0 | 0 | 0.233463 | 0.125984 | 0 |
| *Lb. curvatus* | 0 | 0 | 1.605709 | 0.038911 | 0 | 0 |
| *Lb. coleohominis* | 2.458592 | 0 | 0 | 0 | 0 | 0 |
| *Lb. delbrueckii* | 0 | 0 | 0 | 4.435798 | 1.448819 | 0 |
| *Lb. fermentum* | 2.199793 | 18.41532 | 0 | 7.042802 | 4.692913 | 19.519 |
| *Lb. gallinarum* | 62.06004 | 0.130605 | 0 | 0 | 0 | 0.52283 |
| *Lb. helveticus* | 0.491718 | 0 | 0 | 0.038911 | 18.20472 | 0 |
| *Lb. nagelii* | 0 | 1.175446 | 0 | 0 | 0 | 2.614151 |
| *Lb. nantensis* | 0 | 0 | 0 | 0.194553 | 0.125984 | 0 |
| *Lb. panis* | 10.19669 | 0 | 0 | 0 | 0 | 0 |
| *Lb. plantarum* | 0.621118 | 4.179364 | 8.073149 | 14.16342 | 8.377953 | 8.957825 |
| *Lb. pontis* | 10.86957 | 0 | 0 | 0 | 0 | 0 |
| *Lb. rossiae* | 0 | 0 | 3.122212 | 0.155642 | 0 | 0 |
| *Lb. siliginis* | 0 | 0.17414 | 0.267618 | 0 | 0 | 0.348554 |
| *Lactobacillus sp* | 0.439959 | 0.043535 | 0.111508 | 0 | 0 | 0.209132 |
| *Lb. vaccinostercus* | 0 | 2.655638 | 0.156111 | 0.466926 | 0.062992 | 2.892994 |
| *P. argentinicus* | 0.05176 | 2.786243 | 11.17306 | 2.879377 | 0 | 3.834089 |
| *Leu. citreum* | 0 | 0.914236 | 0.223015 | 0 | 0 | 0.97595 |
| *W. beninensis* | 0.07764 | 0.34828 | 0 | 0 | 0 | 1.394214 |
| *W. confusa* | 0.02588 | 0.696561 | 0 | 0 | 0 | 1.359359 |
| *W. fabalis* | 0 | 0.827166 | 0 | 0 | 0 | 1.045661 |
| *W. fabaria* | 0 | 15.41141 | 0 | 0 | 0 | 15.33635 |
| *W. salipiscis* | 0 | 0 | 0 | 0.272374 | 0.031496 | 0 |
| *Weissella sp* | 0 | 0 | 0.223015 | 0 | 0 | 0 |
| *Lc. lactis* | 0 | 3.569874 | 0 | 0.038911 | 0 | 5.576856 |
| *Lactococcus sp* | 0 | 0.217675 | 0 | 0 | 0 | 0.627396 |
| *Streptococcus sp* | 0 | 0 | 0 | 0 | 0 | 0.069711 |
| *Gemmata sp* | 0 | 0 | 0 | 0 | 0.094488 |  |
| *A. cibinongensis* | 0.07764 | 4.179364 | 0 | 0 | 0 | 5.158592 |
| *A. fabarum* | 0.284679 | 0.391815 | 0.379126 | 0.933852 | 0.15748 | 0.662252 |
| *A. lovaniensis* | 0 | 0 | 2.564674 | 1.595331 | 1.700787 | 0 |
| *A. orientalis* | 0 | 0 | 0.490633 | 2.723735 | 3.685039 | 0 |
| *Sphingomonas sp* | 0 | 0 | 0 | 0 | 0 | 0.034855 |
| *Acetobacter sp* | 0 | 0 | 66.50312 | 64.0856 | 60 | 0 |
| *G. oxydans* | 0 | 0 | 0 | 0.155642 | 0 | 0 |
| *Sphingobium sp* | 0.232919 | 0.522421 | 0.223015 | 0 | 0.409449 | 1.010805 |
| *C. terrigena* | 0 | 0.08707 | 0 | 0 | 0 | 0.034855 |
| *Delftia sp* | 0 | 0 | 0 | 0 | 0 | 0.139421 |
| *E. aerogenes* | 0 | 0.17414 | 0 | 0 | 0 | 0.104566 |
| *Escherichia sp* | 0 | 0.043535 | 0 | 0 | 0 | 0.278843 |
| *P. agglomerans* | 0 | 0 | 0 | 0 | 0 | 0 |
| *Serratia sp* | 0 | 0.043535 | 0 | 0 | 0 | 0.034855 |
| *Acinetobacter sp* | 0 | 0.043535 | 0 | 0 | 0 | 0 |
| *A. ursingii* | 0 | 0.043535 | 0 | 0 | 0 | 0.069711 |
| *Dechloromonas sp* | 0 | 0 | 0.022302 | 0 | 0.031496 | 0.069711 |
| *Pseudomonas sp* | 0 | 0 | 0.066905 | 0 | 0.472441 | 0 |
| *Stenotrophomonas bacterium* | 0 | 0 | 0 | 0 | 0 | 0.069711 |
| *F. aurantia* | 0 | 0.043535 | 0 | 0 | 0 | 0 |
| *Sugarcane phytoplasma* | 9.912008 | 40.35699 | 0 | 0 | 0 | 23.49251 |
|  |  |  |  |  |  |  |
| *no hit* | 0 | 2.394427 | 0.15611 | 0.50584 | 0.34646 | 3.102126 |
